# Urinary Androgens Provide Additional Evidence Related to Metabolism and Are Correlated With Serum Androgens in Girls

**DOI:** 10.1210/jendso/bvad161

**Published:** 2024-01-16

**Authors:** Sasinya N Scott, Marvin Siguencia, Frank Z Stanczyk, Michaela F Hartmann, Stefan A Wudy, Melissa White, Wendy K Chung, Regina M Santella, Mary Beth Terry, Lauren C Houghton

**Affiliations:** Department of Epidemiology, Columbia University Mailman School of Public Health, New York, NY 10032, USA; Department of Epidemiology, Columbia University Mailman School of Public Health, New York, NY 10032, USA; Department of Obstetrics and Gynecology, Keck School of Medicine, University of Southern California, Los Angeles, CA 90033, USA; Steroid Research and Mass Spectrometry Unit, Laboratory of Translational Hormone Analytics in Pediatric Endocrinology, Division of Pediatric Endocrinology & Diabetology, Center of Child and Adolescent Medicine, Justus Liebig University, 35392, Giessen, Germany; Steroid Research and Mass Spectrometry Unit, Laboratory of Translational Hormone Analytics in Pediatric Endocrinology, Division of Pediatric Endocrinology & Diabetology, Center of Child and Adolescent Medicine, Justus Liebig University, 35392, Giessen, Germany; Department of Epidemiology, Columbia University Mailman School of Public Health, New York, NY 10032, USA; Herbert Irving Comprehensive Cancer Center, Columbia University Medical Center, New York, NY 10032, USA; Departments of Pediatrics and Medicine, Columbia University Medical Center, New York, NY 10032, USA; Herbert Irving Comprehensive Cancer Center, Columbia University Medical Center, New York, NY 10032, USA; Department of Environmental Health Sciences, Columbia University Mailman School of Public Health, New York, NY 10032, USA; Department of Epidemiology, Columbia University Mailman School of Public Health, New York, NY 10032, USA; Herbert Irving Comprehensive Cancer Center, Columbia University Medical Center, New York, NY 10032, USA; Department of Epidemiology, Columbia University Mailman School of Public Health, New York, NY 10032, USA; Herbert Irving Comprehensive Cancer Center, Columbia University Medical Center, New York, NY 10032, USA

**Keywords:** urine, serum, androgens, metabolites, puberty, adolescents

## Abstract

**Context:**

Androgen levels are generally measured in serum samples, but urine may be a more feasible option, especially in children, as it is a noninvasive alternative.

**Objective:**

To assess the correlations of 10 urinary androgen metabolites with 4 serum androgens [dehydroepiandrosterone-sulfate (DHEA-S), androstenedione, and total and free testosterone] and assess if their correlations differ by participant characteristics.

**Methods:**

Our study consisted of 44 girls, ages 6-13, who participated in the New York site of the LEGACY Girls Study and had both serum and urine samples collected at the same visit. We performed Pearson's correlation coefficient tests between 4 serum and 10 individual urinary metabolite measures and their sum. We examined the influence of participant characteristics on the magnitude and direction of the correlations.

**Results:**

The summed urinary metabolite measures had the highest correlation with free testosterone in serum (global sum, r = 0.83) and correlated least with DHEA-S in serum (global sum, r = 0.64). The correlation between individual urinary metabolites and serum androgens ranged from 0.08 to 0.84.Two 11-oxygenated urinary metabolites (5α-androstane-3α-ol-11,17-dione5β-androstane-3α,11β-diol-17-one) were weakly correlated with all serum androgens. Participant age, weight, height, waist:hip ratio, and pubic hair growth stage changed the correlations between urinary and serum androgens measures between 10% and 213%.

**Conclusion:**

The sum of urinary androgen metabolites was a good marker of circulating androstenedione, testosterone, and free testosterone. Individual urinary metabolites provide additional information about the metabolic processes of disease development compared to the antecedent serum androgens.

## Background

Elevated levels of circulating androgens in the blood are associated with the development of various health conditions, like polycystic ovary syndrome, insulin resistance, diabetes, high cholesterol, high blood pressure, heart disease, and breast cancer [1-4]. While studies have primarily relied on blood samples to assess androgen levels in developing chronic diseases, urine specimens [5], are easier to collect and noninvasive and may give a similar or more comprehensive measure of androgen production and metabolism. In particular, Remer et al [6] and Wudy et al [7] found that while the body metabolizes and excretes androgens through multiple processes and pathways, urinary androgens reflect adrenarche more reliably than circulating plasma androgens in children and adolescents. Additionally, when collecting a timely defined urinary sample (eg, a 24-hour specimen), androgen metabolites in urine provide reliable estimates of androgen excretion rates (eg, µg/24 hour) and thus are a measure of daily androgen production [8, 9], independent of circadian rhythm. In contrast, a single plasma concentration measurement (ng/mL) is a snapshot at a given point of time. Gas chromatography-mass spectrometry (GC-MS) is one of the new metabolomics platform technologies providing the greatest potential for simultaneously determining a multitude of urinary steroid metabolites, allowing for the delineation of the global steroid metabolome of an individual [10]. It provides a greater opportunity to incorporate comprehensive urinary hormone measures into population-based studies and clinical practice [11].

Age, pubertal stage, menstrual cycle, diet, and diurnal rhythms influence steroid hormone production and metabolic clearance. For example, adrenarche, defined as the rise in the principal adrenal C_19_ steroid dehydroepiandrosterone sulfate (DHEA-S), occurs around the age of 6 to 8 years. It is the hallmark of adrenal production of androgens, while, later in life, the ovaries additionally produce androgens after the activation of the hypothalamic-pituitary-ovarian axis during puberty [12]. Hence, the source of androgen production changes with development [13-15], and the correlation between serum and urinary androgens may also change depending on life stage [16-18]. Therefore, understanding when the correlation between serum androgens and urinary metabolites fluctuates could elucidate when crucial points of disease development occur [19].

Despite the relevance of androgens to many health outcomes across the life course and the need to integrate the steroid metabolome into larger epidemiologic studies, there remains a paucity of data on the utility and expansive capacity of urinary metabolites and their correlations with serum androgen measures. As such, our study aims to establish the viability and detailed array of 10 urinary androgen metabolites identified via GC-MS and their correlations with 4 serum androgens—DHEA-S, androstenedione, and total and free testosterone—in girls before and during puberty from the New York site of the Lessons in Epidemiology and Genetics of Adult Cancer from Youth (LEGACY) Girls Study.

## Methods and Materials

### Study and Participants

**Figure 1.**
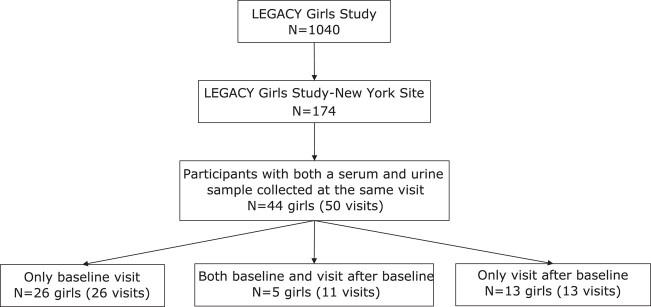
Study participant selection flow chart.

The study sample is a subset of a larger cohort, the LEGACY Girls Study (n = 1040) [20]. The focus of the prospective parent study was to determine the relationship between early-life exposures and intermediate markers of breast cancer risk (eg, pubertal development, breast tissue characteristics) and investigate psychosocial well-being and health behaviors in the context of breast cancer family history. Described elsewhere [21] are the additional details regarding the recruitment of participants and their follow-up. Our inclusion criteria were girls who had both a serum and urine specimen collected at the same visit and had enough additional specimens for our analysis. We determined that 44 girls out of the 174 girls who participated at the New York site of the LEGACY study fit those criteria (Fig. 1).The 44 girls included in our study were between the ages of 6 and 13 [22]; 59% had only a baseline visit (26 visits from 26 girls), 11% had both a baseline visit and 1 or more visits after baseline (11 visits from 5 girls), and 30% (n = 13 girls) had only a visit after baseline (13 visits from 13 girls), for a total of 50 visits across all girls in this study subset. Of the 31 girls with a baseline visit, 71% had not yet started puberty (n = 22) and were between the ages of 6 and 10. At follow-up visits, 63% (n = 12) of girls were pubertal, defined as having 1 or more instances of the start of pubertal development (first occurrence of menstruation (menarche), breast development (thelarche), or pubic hair growth (pubarche)). Puberty data were missing for 4 of the 19 follow-up visits [19].

### Institutional Review Board Approval

Our team obtained Institutional Review Board approval from Columbia University to conduct the study. In addition, all participants and guardians provided written informed consent, and girls provided assent based on institutional standards.

### Biospecimen Collection

We analyzed blood and urine samples provided on the same day during a scheduled study visit. A trained phlebotomist collected blood (15-20 mL) (nonfasting at baseline, fasting at first follow-up, and every 12 months after that) [21]. At the same study visit, participants provided first-morning urine samples. We used stored serum and urine (frozen at −80° C) for these analyses.

### Circulating and Urinary Androgen Measurements

The Reproductive Endocrine Research Laboratory at the University of Southern California Keck School of Medicine in Los Angeles, California, measured 3 serum androgens: DHEA-S, androstenedione, and total testosterone; free testosterone was calculated [23, 24]. The laboratory used a radioimmunoassay (RIA) with preceding organic solvent extraction [ethyl acetate: hexane (3:2)] and Celite column partition chromatography to separate serum testosterone and androstenedione. The 2 androgens were eluted from the column with 10% and 60% toluene in isooctane. An iodinated marker was used in the RIAs [25]. In addition, the laboratory used a direct chemiluminescent immunoassay for measuring DHEA-S concentrations on an Immulite 2000 analyzer (Siemens). This assay method agrees well with liquid-chromatography tandem mass spectrometry [26]. The levels of detection (LOD) for DHEA-S, androstenedione, and testosterone assays were 0.15 μg/mL, 0.03 ng/mL, and 0.02 ng/mL, respectively. The overall intraassay and interassay coefficients of variation (CV) were in the range of 4% to 8% and 9.5% to 12%, respectively. They calculated free testosterone using a validated algorithm [27]. The free testosterone levels have been found to be nearly identical with corresponding values determined by equilibrium dialysis, which is considered to be the gold standard for measuring free testosterone [28].

**Figure 2.**
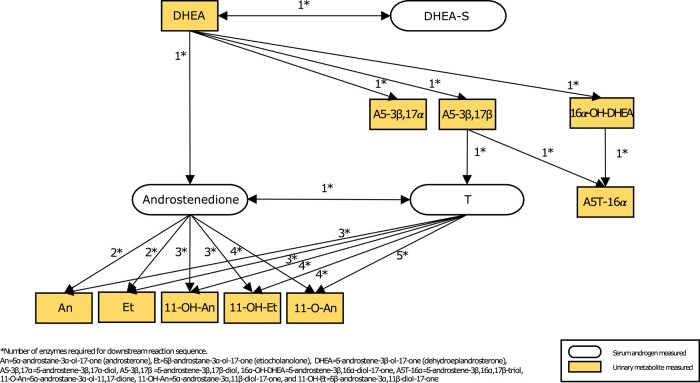
Metabolic relation of the androgens measured in our study population.

The Steroid Research and Mass Spectrometry Unit of the Center of Child and Adolescent Medicine at Justus Liebig University in Germany analyzed urinary metabolites using GC-MS [10, 29]. The GC-MS urinary steroid hormone metabolome assay measured 10 androgen metabolites, including 5α-androstane-3α-ol-17-one [androsterone (An)], 5β-androstane-3α-ol-17-one [etiocholanolone (Et)], 5-androstene-3β-ol-17-one [dehydroepiandrosterone (DHEA)], 5-androstene-3β,17α-diol (A5-3β,17α), 5-androstene-3β,17β-diol (A5-3β,17β), 5-androstene-3β,16α-diol-17-one (16α-OH-DHEA), 5-androstene-3β,16α,17β-triol (A5T-16α), 5α-androstane-3α-ol-11,17-dione (11-O-An), 5α-androstane-3α,11β-diol-17-one (11-OH-An), and 5β-androstane-3α,11β-diol-17-one (11-OH-Et). A detailed list of these 10 urinary metabolites and their abbreviations is described elsewhere [30]. Additionally, they measured creatinine using a Dimension Vista® analyzer (Siemens Healthineers, Eschborn, Germany). As gas chromatography has the greatest separation power for steroid metabolites, GC-MS is currently the method of choice for characterizing the urinary steroid metabolome. The LOD for the urinary metabolites were <3.13 µg/L for An, Et, 11-OH-An, and A5T-16α; < 6.25 µg/L for A5-3β,17α, A5-3β,17β, 11-O-An, and 11-OH-Et; and <12.5 µg/L for DHEA and 16α-OH-DHEA. For all urinary steroids measured, intraassay precision varies between 1.7% (for 17b-Adiol) and 9.5% (for 20α-DHF), and interassay precision ranges between 1.1% (for a-cortol) and 9.5% (for 11-OH-An) [29]. Fig. 2 illustrates the serum and urinary androgens of interest and how they are metabolically related.

### Covariates

We used age (years), weight (kg), height (cm), body mass index (BMI) (kg/m2), percent body fat, and waist:hip ratio from baseline collection. We obtained pubertal stage values from the Pubertal Development Scale (PDS) questionnaire answered by the participants’ guardians at each visit. Guardians rated thelarche and pubarche using the PDS scale 1 to 4, 1: “not yet started,” 2: “barely started,” 3: “definitely started,” 4: “seems complete,” and missing: “I don’t know.” We dichotomized each PDS score as 0 (puberty had not yet started, PDS = 0) and 1 (puberty had started; PDS ≥ 2) [31]. We also collected pubertal development data via guardian-reported Tanner stages but used PDS in this manuscript since we have previously shown that PDS correlates more with hormone concentrations than Tanner stage. To define menarche, guardians reported if their daughter had their first menses and, if so, recalled the age to the nearest half-year [32, 33].

### Statistical Analysis

We calculated within-batch and between-batch CV from blinded pairs of quality control samples in each batch and across batches, respectively. We observed some serum and urinary metabolite measures under the LOD, and the corresponding numbers (n) are displayed in Tables 1 and 2. We evaluated LOD measures with 3 imputation approaches: (1) the LOD, (2) half the value of the LOD, and (3) 0 and censoring the values (removing them from analysis). We detected fluctuations in the mean, median, and range for the varying LOD imputation resolution strategies, so we decided to censor all serum and urinary values under the LOD [34]. Urinary testosterone measures were not included in our analysis because 80% of measures were below the detection limit. To reduce the skewness in our metabolite data, we normalized the distributions by natural log-transforming all urinary metabolite and serum androgen data. To account for urinary dilution at the time of measurement, we divided the urinary metabolite concentrations for an individual by the creatinine value for the same individual [35, 36]. We then used the natural log-transformed creatinine-corrected values for all analyses.

**Table 1.** Demographic and clinical characteristics of the study participants, n = 50 visits for 44 participants

Characteristics	n	Mean (SD)	Column %
Age (years)	50	8.3 (1.97)	100
Weight (kg)	50	34.45 (13.65)	100
Height (cm)	50	134.22 (13.76)	100
BMI (kg/m2)	50	18.61 (4.58)	100
Race/Ethnicity			
Black	6	—	14
Hispanic	16	—	36
White	19	—	43
Other	3	—	7
Menarche (at any visit)*^a^*		—	
Yes	8	—	16
No	39	—	78
Unknown	3	—	6
Breast development (at any visit)*^b^*			
Yes	16	—	32
No	30	—	60
Unknown	4	—	8
Public hair development (at any visit)*^b^*			
Yes	15	—	30
No	31	—	62
Unknown	4	—	8

Abbreviations: BMI, body mass index.

^*a*^Guardians reported if their daughter had their first menses.

^*b*^Pubertal Development Scale values from a questionnaire answered by the participants’ guardian at each visit.

We calculated summed metabolite excretion measures for all metabolites in our study (global sum: An + Et + A5-3β,17α + A5-3β,17β + DHEA + 11-OH-An + 11-O-An + 11-OH-Et + 16α-OH-DHEA + A5T-16α) and for DHEA and its 16-hydroxylated downstream metabolites (DHEA sum: DHEA + 16α-OH-DHEA + and A5T-16α). We assessed mean metabolite concentration differences using paired t-tests. To determine the agreement between serum and urine metabolite measurements, we performed the Pearson's coefficient test and Lin's concordance correlation coefficient. A high Pearson's correlation was defined as r ≥0.75. Lin's concordance correlation coefficient measures precision and accuracy, with the rho-C values ranging from −1, denoting perfect discordance, to +1 for perfect concordance. We assessed the influence of participant characteristics on the magnitude and direction of the relationship between serum and urine metabolites using the percent difference of parameter estimates from linear regressions adjusted for each covariate individually (age, weight, height, BMI, percent body fat, waist:hip ratio, menarche, thelarche, and pubarche). Additionally, 5 participants in our sample population had more than 1 visit with both serum and urine samples included. Therefore, to consider the correlation of within-subject data for these 5 participants, we used generalized estimating equations for all serum and urine comparisons and adjusted for the same covariates as the linear regression. We standardized the regression parameters for comparison with Pearson's correlations. We conducted all analyses using SAS statistical software (version 9.4, SAS Institute Inc., Durham, NC, USA).

## Results

The demographic and clinical characteristics and androgen measurements for the study participants are shown in Table 1. Of the 44 girls selected, 43% (n = 19) were White, 36% (n = 16) were Hispanic, and 14% (n = 6) were Black. The mean age, weight, height, and BMI were 8.3 years, 34.4 kg, 134.2 cm, and 18.6 kg/m^3^, in the order stated. For all steroid hormones measured in serum, the within- and between-batch CV (%) ranges were 0.4% to 16.8% and 4.1% to 16.1%, respectively. The within- and between-batch CV (%) ranges for the urinary metabolites were 0% to 11.1% and 6.7% to 9.9%, respectively.

### Urinary Metabolites

Tables 2 to 5 present the descriptive measures and correlations for each individual urinary androgen, summary measures, and circulating androgen [DHEA-S (Table 2), androstenedione (Table 3), testosterone (Table 4), and free-testosterone (Table 5)]. The most abundant urinary metabolites were An (mean = 4.3 µg/g creatinine) and Et (mean = 3.7 µg/g creatinine). The lowest urinary C_19_-steroids were A5-3β,17α (mean = 0.2 µg/g creatinine) and A5-3β,17β (mean = 0.2 µg/g creatinine). Additionally, An had the highest Pearson correlations (r) with all serum androgens (DHEA-S: r = 0.62, androstenedione: r = 0.77, testosterone: r = 0.82, and free testosterone: r = 0.84), while urinary 11-O-An and 11-OH-Et were the least correlated with serum androgens, ranging from 0.07 to 0.30.

**Table 2.** Comparing serum androgen DHEA-S and urinary metabolites descriptive measures and correlations

Analyte	n	CV% range	Median	Range		Mean	Pearson's correlation coefficient (rho-C)	
				Min	Max			*P*-value
DHEA-S (µg/mL)*^a^*	36	1.61-8.90	0.68	0.15	1.97	0.71	—	—
Global sum (µg/g creatinine)	36	—	—	—	—	—	0.64	<.01
DHEA sum (µg/g creatinine)	36	—	—	—	—	—	0.54	<.01
An (µg/g creatinine)	36	0.97-4.92	3.5	0.48	18.2	4.24	0.62	<.01
Et (µg/g creatinine)	36	0.17-4.97	3.11	0.32	13.7	3.67	0.61	<.01
DHEA (µg/g creatinine)	34	1.37-5.75	0.31	0	4.52	0.55	0.37	0.03
A5-3β,17α (µg/g creatinine)	35	0.70-7.74	0.15	0	0.5	0.16	0.55	<.01
A5-3β,17β (µg/g creatinine)	32	1.30-11.1	0.16	0	0.64	0.15	0.54	.02
16α-OH-DHEA (µg/g creatinine)	31	0.97-3.00	0.6	0	11.1	0.97	0.41	.02
A5T-16α (µg/g creatinine)	36	0.87-5.86	0.48	0	7.33	0.75	0.53	<.01
11-O-An (µg/g creatinine)	36	0.27-4.76	0.28	0.1	0.85	0.33	0.07	.67
11-OH-An (µg/g creatinine)	36	0.63-4.58	1.51	0.43	6.23	1.93	0.31	.06
11-OH-Et (µg/g creatinine)	36	0.03-4.74	0.98	0.1	5.92	1.49	0.08	.66

Abbreviations: 11-O-An, 5α-androstane-3α-ol-11,17-dione; 11-OH-An, 5α-androstane-3α,11β-diol-17-one; 11-OH-Et, 5β-androstane-3α,11β-diol-17-one;16α-OH-DHEA, 16α-diol-17-one; A5-3β,17α, 5-androstene-3β,17α-diol; A5T-16α, 5-androstene-3β,16α,17β-triol; An, androsterone; CV, coefficient of variation; DHEA, dehydroepiandrosterone; DHEA-S, dehydroepiandrosterone-sulfate; Et, etiocholanolone.

^*a*^Serum androgen, global sum: An, Et, DHEA, A5-3β,17α, A5-3β,17β, 16α-OH-DHEA, A5T-16α, 11-O-An, 11-OH-An, 11-OH-Et; DHEA sum: DHEA, 16α-OH-DHEA, and A5T-16α.

**Table 3.** Comparing serum androgen androstenedione and urinary metabolites descriptive measures and correlations

Analyte	n	CV% range	Median	Range		Mean	Pearson's correlation coefficient	
				Min	Max			*P*-value
Androstenedione (ng/mL)*^a^*	50	1.36-7.29	0.22	0.03	1.17	0.33	—	—
Global sum (µg/g creatinine)	50	—	—	—	—	—	0.76	<.01
DHEA sum (µg/g creatinine)	49	—	—	—	—	—	0.62	<.01
An (µg/g creatinine)	50	0.97-4.92	3.5	0.48	18.2	4.24	0.77	<.01
Et (µg/g creatinine)	50	0.17-4.97	3.11	0.32	13.7	3.67	0.77	<.01
DHEA (µg/g creatinine)	36	1.37-5.75	0.31	0	4.52	0.55	0.19	.26
A5-3β,17α (µg/g creatinine)	39	0.70-7.74	0.15	0	0.5	0.16	0.58	.01
A5-3β,17β (µg/g creatinine)	34	1.30-11.1	0.16	0	0.64	0.15	0.49	<.01
16α-OH-DHEA (µg/g creatinine)	33	0.97-3.00	0.6	0	11.1	0.97	0.39	.02
A5T-16α (µg/g creatinine)	49	0.87-5.86	0.48	0	7.33	0.75	0.63	<.01
11-O-An (µg/g creatinine)	50	0.27-4.76	0.28	0.1	0.85	0.33	0.27	.06
11-OH-An (µg/g creatinine)	50	0.63-4.58	1.51	0.43	6.23	1.93	0.49	<.01
11-OH-Et (µg/g creatinine)	50	0.03-4.74	0.98	0.1	5.92	1.49	0.24	.09

Abbreviations: 11-O-An, 5α-androstane-3α-ol-11,17-dione; 11-OH-An, 5α-androstane-3α,11β-diol-17-one; 11-OH-Et, 5β-androstane-3α,11β-diol-17-one;16α-OH-DHEA, 16α-diol-17-one; A5-3β,17α, 5-androstene-3β,17α-diol; A5T-16α, 5-androstene-3β,16α,17β-triol; An, androsterone; CV, coefficient of variation; DHEA, dehydroepiandrosterone; DHEA-S, dehydroepiandrosterone-sulfate; Et, etiocholanolone.

^*a*^Serum androgen, global sum: An, Et, DHEA, A5-3β,17α, A5-3β,17β, 16α-OH-DHEA, A5T-16α, 11-O-An, 11-OH-An, 11-OH-Et; DHEA sum: DHEA, 16α-OH-DHEA, and A5T-16α.

**Table 4.** Comparing serum androgen testosterone and urinary metabolites descriptive measures and correlations

Analyte	n	CV% range	Median	Range		Mean	Pearson's correlation coefficient	
				Min	Max			*P*-value
Testosterone (ng/mL)*^a^*	50	2.14-12.2	0.07	0.02	0.51	0.1	—	—
Global sum (µg/g creatinine)	50	—	—	—	—	—	0.80	<.01
DHEA sum (µg/g creatinine)	49	—	—	—	—	—	0.64	<.01
An (µg/g creatinine)	50	0.97-4.92	3.5	0.48	18.2	4.24	0.82	<.01
Et (µg/g creatinine)	50	0.17-4.97	3.11	0.32	13.7	3.67	0.80	<.01
DHEA (µg/g creatinine)	36	1.37-5.75	0.31	0	4.52	0.55	0.18	.30
A5-3β,17α (µg/g creatinine)	39	0.70-7.74	0.15	0	0.5	0.16	0.60	<.01
A5-3β,17β (µg/g creatinine)	34	1.30-11.1	0.16	0	0.64	0.15	0.47	<.01
16α-OH-DHEA (µg/g creatinine)	33	0.97-3.00	0.6	0	11.1	0.97	0.38	.03
A5T-16α (µg/g creatinine)	49	0.87-5.86	0.48	0	7.33	0.75	0.65	<.01
11-O-An (µg/g creatinine)	50	0.27-4.76	0.28	0.1	0.85	0.33	0.28	.05
11-OH-An (µg/g creatinine)	50	0.63-4.58	1.51	0.43	6.23	1.93	0.54	<.01
11-OH-Et (µg/g creatinine)	50	0.03-4.74	0.98	0.1	5.92	1.49	0.26	.07

Abbreviations: 11-O-An, 5α-androstane-3α-ol-11,17-dione; 11-OH-An, 5α-androstane-3α,11β-diol-17-one; 11-OH-Et, 5β-androstane-3α,11β-diol-17-one;16α-OH-DHEA, 16α-diol-17-one; A5-3β,17α, 5-androstene-3β,17α-diol; A5T-16α, 5-androstene-3β,16α,17β-triol; An, androsterone; CV, coefficient of variation; DHEA, dehydroepiandrosterone; DHEA-S, dehydroepiandrosterone-sulfate; Et, etiocholanolone.

^*a*^Serum androgen, global sum: An, Et, DHEA, A5-3β,17α, A5-3β,17β, 16α-OH-DHEA, A5T-16α, 11-O-An, 11-OH-An, 11-OH-Et; DHEA sum: DHEA, 16α-OH-DHEA, and A5T-16α.

**Table 5.** Comparing serum androgen free testosterone and urinary metabolites descriptive measures and correlations

Analyte	n	CV% range	Median	Range		Mean	Pearson's correlation coefficient	
				Min	Max			*P*-value
Free testosterone (ng/mL)*^a^*	50	0.49-16.7	0	0	0.01	0.002	—	—
Global sum (µg/g creatinine)	50	—	—	—	—	—	0.82	<.01
DHEA sum (µg/g creatinine)	49	—	—	—	—	—	0.71	<.01
An (µg/g creatinine)	50	0.97-4.92	3.5	0.48	18.2	4.24	0.84	<.01
Et (µg/g creatinine)	50	0.17-4.97	3.11	0.32	13.7	3.67	0.82	<.01
DHEA (µg/g creatinine)	36	1.37-5.75	0.31	0	4.52	0.55	0.29	.08
A5-3β,17α (µg/g creatinine)	39	0.70-7.74	0.15	0	0.5	0.16	0.67	<.01
A5-3β,17β (µg/g creatinine)	34	1.30-11.1	0.16	0	0.64	0.15	0.60	<.01
16α-OH-DHEA (µg/g creatinine)	33	0.97-3.00	0.6	0	11.1	0.97	0.46	<.01
A5T-16α (µg/g creatinine)	49	0.87-5.86	0.48	0	7.33	0.75	0.70	<.01
11-O-An (µg/g creatinine)	50	0.27-4.76	0.28	0.1	0.85	0.33	0.30	.04
11-OH-An (µg/g creatinine)	50	0.63-4.58	1.51	0.43	6.23	1.93	0.58	<.01
11-OH-Et (µg/g creatinine)	50	0.03-4.74	0.98	0.1	5.92	1.49	0.22	.13

Abbreviations: 11-O-An, 5α-androstane-3α-ol-11,17-dione; 11-OH-An, 5α-androstane-3α,11β-diol-17-one; 11-OH-Et, 5β-androstane-3α,11β-diol-17-one;16α-OH-DHEA, 16α-diol-17-one; A5-3β,17α, 5-androstene-3β,17α-diol; A5T-16α, 5-androstene-3β,16α,17β-triol; An, androsterone; CV, coefficient of variation; DHEA, dehydroepiandrosterone; DHEA-S, dehydroepiandrosterone-sulfate; Et, etiocholanolone.

^*a*^Serum androgen, global sum: An, Et, DHEA, A5-3β,17α, A5-3β,17β, 16α-OH-DHEA, A5T-16α, 11-O-An, 11-OH-An, 11-OH-Et; DHEA sum: DHEA, 16α-OH-DHEA, and A5T-16α.

### Serum Androgens

DHEA-S was the most abundant serum androgen (mean = 0.71 µg/mL) but the least correlated with the summary urinary measures (global sum: r = 0.64 and DHEA sum: r = 0.54) compared to the other serum androgens. Correlations between DHEA-S and most urinary metabolites measured, except 11-O-An (r = 0.07), and 11-OH-Et (r = 0.08), ranged between 0.31 and 0.62. Androstenedione was highly correlated with the global sum (r = 0.76) but with the tightest range of correlations observed, ranging between r = 0.24 and 0.77, with An (r = 0.77) and Et (r = 0.77) being the highest values. Testosterone was also highly correlated with global sum (r = 0.8). Free testosterone was the least abundant serum androgen (mean = 0.002 ng/mL) but the most correlated with both urinary metabolite summary measures (global sum: r = 0.83 and DHEA sum: r = 0.71). It had the highest correlations observed (An: r = 0.84 and Et: r = 0.82) but the broadest range of correlations observed between the highest (An: r = 0.84) and lowest urinary metabolites (11-OH-Et: r = 0.22). There were no apparent patterns between the strength of correlation and the number of enzymatic steps from precursor androgens to metabolites as illustrated in Fig. 2.

### Impact of Demographic and Clinical Factors on the Correlation Between Serum and Urinary Androgen Metabolites

**Figure 3.**
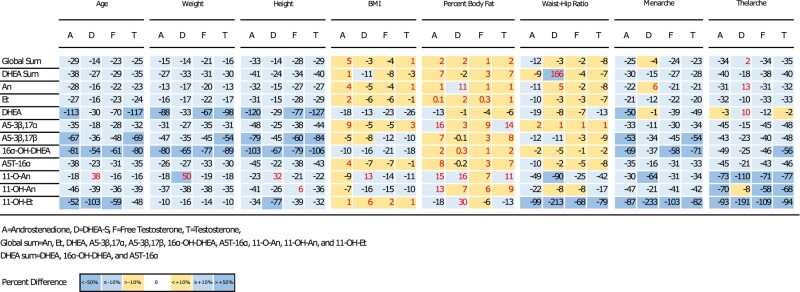
Percent difference of linear regression estimates for the comparison between serum androgens and urinary metabolites while adjusting for participant characteristics.

Figure 3 summarizes the impact of participant characteristics (age, weight, height, BMI, percent body fat, waist:hip ratio, menarche, thelarche, and pubarche) on the correlation between serum androgens and urinary metabolites. Age, weight, height, and pubarche changed the correlations between the serum androgens and most urinary metabolites, including the summed urinary measures, by more than 10%. These changes in correlation were most noticeable for A5-3β,17β, 16α-OH-DHEA, and 11-OH-Et (52-117% change). Markers of adiposity, which included BMI and percent body fat, induced very little change (less than 10%) between serum androgen and most urinary metabolites correlations. However, the waist:hip ratio predictor had the widest range of influence on the correlations between serum and urinary androgens. For example, it changed the correlation between DHEA sum and DHEA-S by 166% and between all 4 serum androgens and 11-OH-Et correlations by as much as 213%.

## Discussion

This study reveals that urinary steroids reflect active serum hormone metabolites. A proportional relationship exists between the global sum of excreted urinary androgen metabolites and serum androstenedione and total and free testosterone. Notably, free testosterone, a biologically active, unbound form of testosterone, had the strongest correlation with the excreted urinary metabolites, underscoring that the sum of urinary steroids reflects biologically active androgens. Further, individual urinary metabolites that were highly correlated with serum androgens are also good proxies for upstream serum androgens. For example, urinary An reflected serum levels of androstenedione and free and total testosterone. Our results also illustrate that age, weight, height, and pubertal benchmarks, such as pubarche, explained some of the variance in the correlation between serum androgens and urinary metabolites. In contrast, adiposity markers, BMI, and percent body fat had little effect on those correlations, apart from waist:hip ratio. Taken together, these findings can help inform the design of future population-based studies using urinary androgens by suggesting which metabolites to measure and guiding the selection of covariates to include in models.

In population health, researchers rely on serum samples to assess androgen levels concerning chronic disease onset, despite urine being a more accessible and advantageous option. In clinical settings, urine is used as a diagnostic tool for steroid profiling for adrenal disorders [29], like postoperative recurrence detection for adrenocortical carcinoma [37], and for monitoring adrenal disorders such as congenital adrenal hyperplasia [38]. Coupled with the ease of sample handling and noninvasive and convenient collection of repeat specimens, Newman et al found that liquid and dried urine were good representatives for serum testing for hormones such as estradiol, progesterone, and cortisol [11, 39, 40]. In line with previous observations [41], we found that our summed urinary metabolite measures were more highly correlated with serum androstenedione and free and total testosterone than individual urinary metabolite measures since the summed urinary metabolite measures comprise metabolized and excreted products of the serum androgens. Given these results, first-morning urine should be considered a valid substance for quantifying urinary androgen metabolite concentrations in children and capturing the analogous information in the steroid metabolome during the pubertal window. The ease of collecting first-morning urine vs 24-hour collection makes this a more feasible option, especially when conducting research with children.

The sum of urinary androgen metabolites is more proportionate to serum androgens than when individual metabolites were compared with serum androgens. However, by measuring more individual metabolites in urine, we can draw more conclusions regarding enzymes involved in androgen metabolism when focusing on single metabolites. For example, 2 of the 11-oxygenated urinary metabolites (11-O-An and 11-OH-Et) reflect enzymatic processes that are not reflected in the precursor serum androgens we measured. Such downstream urinary metabolites may be target biomarkers for studies interested in androgen metabolism rather than androgen production. The 11-oxygenated androgens have been implicated in polycystic ovary syndrome and breast cancer risk in adults [42, 43].

Previous studies show that BMI affects the correlations between serum estrogens and estrogen metabolites in adults [44]. However, we observed that BMI and percent body fat had minor effects on the correlations between serum androgens and urinary androgen metabolites. While fat tissue can produce both androgens and estrogens, the range of BMI in our study sample may not capture the level of obesity necessary to affect androgen levels. BMI is generally regarded as a less robust indicator of body fat and reflects a different construct to waist:hip ratio, which is a more precise estimation of abdominal fat. Similarly, we used bioimpedance to measure percent body fat, which is not as precise as DXA scans; therefore, its lack of impact on the correlation may be due to precision of measurement. Additionally, we found that factors like age, weight, height, and pubarche changed the correlations between serum androgens and urinary androgen metabolites by as little as 10% and as much as 233% in girls ages 6 to 13. This influence is biologically plausible because androgen production starts around adrenarche (ages 6-8) and continues to increase with age. Once pubarche is initiated, there is further production of androgens from the gonads. Therefore, the variance between serum and urine measures could be explained by differences in androgen production according to age, pubertal stage, and body size. Some of the variance could also be due to a time lag in serum and urinary measures.

A limitation of our study is that most adolescent urinary testosterone concentrations (n = 40, 80%) were below the limit of detection of the GC-MS assay. As a result, we could not compare testosterone between the serum and urine samples. Another limitation was that we observed lower concentrations for multiple urinary metabolites. For instance, of the total of measures (n = 700), 8.4% (n = 59) were below the LOD. There were 14 samples below the LOD for serum DHEAS, but no samples were below the LOD for other serum androgens. Regarding the urinary metabolites, only 4 had samples below the LOD. We observed that 11 measures of A5-3β,17α, 16 measures of A5-3β,17β, 17 measures of 16α-OH-DHEA, and 1 measure of A5T-16α were under the LOD. Since androgen metabolite production increases with puberty, we anticipated possible low concentrations of metabolites due to the prepubertal nature of most girls in our cohort. Additionally, serum concentrations of androgens reflect instantaneous levels in circulation, whereas urinary levels adjusted for creatinine reflect excreted levels at a standardized rate. Creatinine adjustments, however, cannot overcome the diurnal variation in first-morning urines, which, compared to 24-hour urines, is a limitation of our study but a strength with regard to study feasibility in field settings. The strength of our study includes the high sensitivity, specificity, and reproducibility of both urinary and serum assays, which allowed close to a 10% CV range for within-batch and less than 20% CV range for between-batch, which is the standard range for conventional RIAs and GC-MS assays. Furthermore, since the CVs are relatively similar between serum and urine, the measurement error is nondifferential.

## Conclusion

Our results support using urinary androgen metabolite measures for large epidemiological studies in girls. We established that urinary androgen metabolite sums are a good indicator of circulating serum androstenedione and total and free testosterone. The findings from our study are encouraging for future studies interested in using single urinary metabolite concentrations to detect active hormonal processes independently associated with disease development and different health outcomes among girls.

## Data Availability

Original data generated and analyzed during this study are included in this published article or in the data repositories listed in References.
